# Proving of Convallaria Majalis

**Published:** 1885-08

**Authors:** Irvin J. Lane

**Affiliations:** Sing Sing, N. Y.


### PROVING OF CONVALLARIA MAJALIS.

IRVIN J. LANE, M. D., SING SING, N. Y.

(*Continued from page 210.*)

VERIFICATIONS.—March 31st I was called to see Mrs. P——, æt. thirty-two. General health good; has had six children.

Her menses stopped January 21st. On the 23d she went out in the evening and paraded around the street with the Salvation Army and got wet and cold. Since then she has had no signs of the menses returning. She was quite certain she was not pregnant, although she had nausea, but could not vomit except at times, especially in the morning and at about 11 A. M., but she said she did not feel as she did when she was pregnant.

For the past three weeks she had had no appetite. Thirst for cold water, but drinking too much would cause nausea. Great prostration. Collection of nasty-tasting water in the mouth. Sore feeling across the hypogastric region. Aching in the back and sides. Restless sleep, especially after midnight. Feels cold all the while, except for the past few days she has had a fever commencing between 3 and 4 P. M., lasting until evening, followed by perspiration, perspiring a great deal nights. Vertigo followed by headache. After eating she feels faint, with a heavy pain in the stomach as of a weight.

I prescribed at different times, up to April 24th, Ars., Lyc., Nux vom., Puls., Sul., Ipec., Apis., Canth. as the symptoms seemed to indicate, but none of them had the desired effect.

April 24th, 4.45 P. M.—Felt very well yesterday A. M. except nausea, but could not vomit. Prostration, thirst, and no appetite. About 1 P. M. the headache and backache commenced quite severe. Chill commenced about 5 P. M. and lasted about an hour and a half, followed by a fever, which lasted all night. No sweat, followed at 8 A. M. April 24th by another chill and fever, which lasted until 12.30 P. M. The chills are preceded by yawning and sleepiness, headache, backache; nails will begin to turn purple; then the chill will commence in the hands and arms, then the feet and legs, and spread all over the body. Thirsty, drinking cold water or lemonade. She would sit near the stove and have a hot fire, but that would not make much difference. Dysuria, with frequent calls to micturate and passage of only a small amount of urine. The urine stained the chamber dark colored, which it was very difficult to wash off.

The urinary difficulty continued not only during the chill and fever but during the apyrexia also.

The headache was different each day, but the same on alternate days. One day the headache would be of a dull character, the next day the pains would be sharp. The days she would have the dull headache, there would be a heavy, dull, aching pain in both temples and vertex; sometimes the pains would extend to the right internal ear, and cause the ear to ache. The above headache was aggravated when walking around, at night, and when lying down, and ameliorated by tying a bandage around the head. The next day the headache would come on suddenly by a sharp pain, starting from the outer side of the right eye and extending to the vertex. This pain would continue for an hour or more, passing off suddenly in a dull, aching pain through both temples. During the sharp pain in the head she had to lie down and keep very quiet, as the least motion, noise, or even when she would speak would aggravate the headache. Has a faint feeling in the stomach, with desire for something to eat, but when she would try to eat it would cause nausea and she would have to stop. Aversion to anything sweet or sour, to potatoes or meat, except ham; after eating a small piece of ham it caused her a great deal of distress in the stomach; aversion to tea or coffee; could not bear even the smell of tea, which she always liked. All she cared for was crackers, bread and butter, ham, oranges, apples, lemonade, and cold water. I prescribed Convallaria majalis $^{30}$ (B. & T.) every two hours.

April 25th.—When I called I found my patient at the neighbors, "making a call." When she came in she said: "I have not got my head tied up to-day, and have been to work; have done my ironing, sweeping, and other house work" (which she had not been able to do before in about seven weeks). She said that about one hour after taking the first dose of the medicine her menses came on but were scanty; the blood was light colored and thicker than common; the flow continued until she went to bed; then she soon fell asleep and slept well all night, which was the first good night's rest she had had in over a month; she always dreaded when it came night on account of the sleeplessness. Has had no headache since about 6 P. M. yesterday, which was the first she had been free from the headache for a whole day in over six weeks.

This morning, when she got up, she says she felt "well," with the exceptions of a backache through the lumbar region and sharp pains across the hypogastric region, just as she always has before the menses appear. The menses stopped during the night. Has had no nausea to-day. Micturition has caused no pain. Urine normal in color and quantity to-day, but was very profuse last night; she says that she made nearly a chamberful during the night. Appetite not very good yet, but she feels stronger. Placebo every two hours.

April 26th.—Has had no backache, headache, or pain across the abdomen; has had some nausea during the day. Sore throat, which is not swollen and only slightly inflamed; sore pain on both sides when swallowing. Placebo.

April 28th.—The sore throat disappeared quite suddenly about noon yesterday. On April 26th it rained nearly all day, and the air was very chilly and damp. She had been used to having the room very warm and close, even in dry, warm weather, but on the above day she seemed to think she was getting well too fast, so she let the fire go out and kept the doors and windows open, so it became very damp and chilly in the room. On the 27th she had a cold so that she coughed quite hard; pain in region of the stomach when coughing, with yellow expectoration. At about 2 P. M. a fever commenced which lasted until she went to bed (8 P. M.). Dull headache all day. To-day she has had a feeling of nausea, with vomiting once in the morning after getting up and once about 1 P. M. Had a slight chill about 12 M., which lasted only a few minutes, and was followed by a fever, which lasted the most of the afternoon. Dull headache all day, appetite poor. ℞ Convallaria majalis $^{30}$ every two hours.

April 29th.—Feels better than for the past two days. Nausea with vomiting immediately after eating or drinking. Feels hungry and faint at the stomach, but cannot eat on account of the vomiting. Has coughed quite frequently to-day, but more last night. Expectoration thick and yellow; pain in region of stomach when coughing. Dull headache this forenoon. Feels dull and sleepy. ℞ placebo. I continued the placebo up to May 11th and she gradually gained so that on the above date she did not complain in any way except of the nausea, which was better.

May 15th.—Saw patient again to-day. On 11th instant I gave her Ipecac $^{30}$ every two hours. After taking the second dose the nausea disappeared and she has not complained since. She did her washing on the 12th and has attended to all her work since.

CASE No. II.—C. E. Lane, M. D., had a sore throat for over a week. The symptoms were as follows: "Throat was sore and burning, and his eyes burned almost as if there was sand in them, but no redness or discharge from the eyes, no redness or swelling in the throat, but the back part of the throat was covered with tough, stringy mucus. Weak feeling in the side, especially around the heart, aggravated by the least exertion. Pulse 55 while exercising. Temperature about a degree below normal. Very nervous and cross. Urine deposited a red sediment like brick dust; looked thick and dark colored when first passed; after standing looked normal in color and clear, except the red sediment." I prescribed Convallaria majalis [30] for the throat symptoms, as they were the only symptoms told me at the time. I do not know that Convallaria has ever caused the symptoms of the throat as above referred to, but I prescribed it from the throat symptoms of Case No. I, which I thought was an aggravation caused by Convallaria. The medicine was sent and prescribed by my sister, who writes me a few days later as follows: "I gave C—— the Convallaria, one dose at night; he did not feel very much better, so the next morning I medicated some pellets and told him to take a dose every three hours. The next morning he was better and I have not heard any trouble about his throat since."

Were the throat symptoms of Case No. I an aggravation by Convallaria, or was the whole case cured by the remedy being indicated by some other symptom?

C. E. Lane, M. D., reports a case of a woman during labor. The pains were weak, and *she would go to sleep between the pains.* He gave Convallaria low, which relieved the above symptom, and the pains came on more forcibly. (See HOMŒOPATHIC PHYSICIAN, Vol. IV, p. 124.)

I have had very good success with Convallaria in a number of cases of weak heart action. (See HOMŒOPATHIC PHYSICIAN, Vol. IV, p. 124.)

